# Does the first generic exclusivity system provide an economic incentive for early generic entrance under the patent linkage system?

**DOI:** 10.3389/fpubh.2023.1120729

**Published:** 2023-08-03

**Authors:** Kyung-Bok Son

**Affiliations:** College of Pharmacy, Hanyang University, Ansan, Gyeonggi-do, Republic of Korea

**Keywords:** first generic exclusivity, patent linkage system, incentive for early generic entry, pharmaceutical market, South Korea

## Abstract

**Introduction:**

A period of exclusivity for the first generics, as part of the patent linkage system, was established in South Korea to provide an economic incentive for early generic entry. This study describes the dynamics of generic penetration and assesses the first mover market share advantages under the patent linkage system.

**Methods:**

Pairs of originators and their corresponding generics granted the first generic exclusivity from 2015 to 2020 were identified. We categorized generics into first movers and latecomers, described the penetration curves of generics, and estimated the saturated market share of generics, first movers, and latecomers. Volume-based monthly prescriptions were used to describe the generics’ penetration curves. A logistic growth model was adopted to estimate the saturated market shares of generics.

**Results:**

We identified 28 pairs of originators and generics, presented penetration curves, and estimated generics market shares. The saturated market share of generics was 30%, and the time to saturation was approximately 33 months. The shapes of penetration varied by nationality, route, and number of generics. The existence of latecomers was associated with the decreased penetration speed over time and decreased market share of generics. However, the first mover market share advantages or latecomers’ disadvantages were consistently observed.

**Conclusion:**

The generic uptake in South Korea is delayed, limited, and context-dependent. However, first generics’ market share advantage suggests that a period of exclusivity, as part of the patent linkage system, could provide an economic incentive for early generic entrance.

## Introduction

The patent linkage system indicates a conditional relationship between the patent of an originator and the market approval of its corresponding generic drug (1). South Korea introduced the linkage system in 2012 after signing the Korea-United States Free Trade Agreement (KORUS) (2). The system in South Korea includes four components—the patent list, the notification process, the stay of market approval for generics, and the nine-month of exclusivity for the first generic entrant (2, 3). The South Korean government introduced a period of exclusivity as a part of the patent linkage system. However, this exclusivity for the first generic is not mandated by the KORUS.

Concerns about delayed generics entry and the high prices of originators that result from their monopoly would have arisen under the patent linkage system (4–6). Empirically, the patent linkage system in the United States effectively delayed generic entry and extended monopoly through a stay of market approval for generics (7). The exclusivity for the first generics, which counters a stay of market approval for generics, was established to provide an economic incentive for generic manufacturers to challenge the validity of the patents of originators and to enter into the generic markets earlier (3). Any manufacturer in South Korea who submitted the dossiers of generics to the regulatory authority and obtained a favorable decision from the patent court could be granted a nine-month exclusivity (8).

Understanding generic market dynamics and assessing first mover advantage is essential to designing the exclusivity for the first generic as part of the patent linkage system. However, evidence describing the generic market and supporting first-mover market advantage is lacking. This study describes the dynamics of generic penetration immediately after the first generic entrance and assesses first mover market share advantages under the patent linkage system. To this end, we categorized generics into first movers and latecomers, described the penetration curves of generics, and estimated the saturated market share of generics, including first movers and latecomers. Findings from this study provide evidence of the dynamics of generic markets and the rationale of exclusivity for the first generics as a valid component of the patent linkage system.

## Methods

### Study design

Given the first generic exclusivity system in South Korea, generics were categorized into first movers and latecomers based on the date of their marketing approval. Generics that were granted the first generic exclusivity were defined as first movers. First movers were granted a nine-month market exclusivity following the first generic exclusivity system, which we intentionally leveraged for the analysis. Latecomers were defined as generics that had been granted marketing approval 9 months after the first generic approval date.

Supplementary File S1 depicts the study flowchart. First, this study identifies pairs of originators and their generics that were granted the first generic exclusivity from March 2015 to December 2020. The first generic exclusivity system was introduced in March 2015 (3). The Ministry of Food and Drug Safety disclose the information on its website (9). Second, pairs of originators and their corresponding generics with periods of observation of fewer than 12 months were excluded from the analysis. Generic penetration reaches saturation at two and a half years from the date of the first generic entrant (10). Third, pairs of originators and generics with issues in patenting or in defining eligible markets were excluded. Patent issues affecting generics could prevent physicians from prescribing generics to their patients. Estimating the market penetration of those generics for which it is not easy to define the market might not be reasonable.

### Patient and public involvement

Patients and the public were not involved in the design, conduct, reporting, or dissemination of this study.

### Variables

The dependent variable is the market share of generics. The originators’ and generics’ monthly prescription volumes were obtained from the Health Insurance and Review and Assessment Service. The market share of the generics was calculated by dividing the number of prescribed generics by the total number of prescribed drugs. The market share of the first generics was calculated by dividing the number of prescribed first generics by the total number of prescribed drugs. The variable was presented beginning with the month when the first generic entered the market.

The pairs of originators and generics were categorized according to four factors: *latecomers, nationality, route,* and *number of generics*. *Latecomer* indicates the existence of latecomers in the originator-generic pairs, which are then categorized into groups without and with latecomers. *Nationality* refers to the market approval holders for originators, classified into domestic and overseas manufacturers. A *route* indicates the route originators are administered, categorized into oral (tablets and capsules) and other forms (such as injections). The *number of generics* indicates the total number of generic brands, which is categorized into a five or fewer generics group and a six or more generics group.

### Statistical analysis

Two types of analysis were used: descriptive analysis and logistic growth models. For descriptive analysis, we described the market dynamics using the penetration curves of the generics. The penetration curves were presented separately according to the four factors. The vertical axis of the curves represents the market share of generics, while the horizontal axis represents the month after the first generic entered the market. These curves can determine the saturated market share of generics, the time to saturated market share, and the speed of generic penetration.

The penetration curves of generics look like a logistical function. The penetration is initially exponential but slows down as saturation is approached. We adopted a logistic growth model using nonlinear least squares to estimate the market share of all generics and first generics, as shown in Equation 1 (11, 12). In the visual assessment of the model assumptions, we found that the residuals were approximately normally distributed. The market share of latecomers was obtained by subtracting all generics’ market share from the first generics’ market share. In the logistic growth model, the dependent variable is the market share of generics, and the independent variable is the months since the entrance of the first generic. Three parameters are noteworthy. Parameter β_1_ is associated with the limiting value or asymptote, β_2_ is associated with the initial value of the market share, and β_3_ is associated with the growth rate, which describes how quickly the dependent variable approaches the limiting value. We reported the first parameters to present the estimated generics’ market share.

Market Share = $\frac{\beta_{1}}{1+\exp\left(-\left(\beta_{2}+\beta_{3}*Month\right)\right)}\left(1\right)$ .

Data management and analysis were performed using R statistical software (version 4.1.3). The nonlinear least squares function in the “car” package was used to estimate the saturated generics’ market share. Two-sided *p*-values of less than 0.05 determined statistical significance.

## Results

### Investigated originator-generic pairs

We identified 28 pairs of originators and their corresponding generics. Table 1 presents the characteristics of the pairs grouped by the presence of latecomers. Approximately half (13 pairs) were categorized into the group without latecomers, and the remaining half (15 pairs) were classified into the group containing latecomers. Of the 13 pairs without latecomers, six (46%) originated from domestic manufacturers, five (38%) were in oral forms, and 13 (100%) included five or fewer generics. Similarly, of the 15 pairs with latecomers, nine (60%) originated from domestic manufacturers, 13 (87%) were in oral forms, and 11 (73%) included six or more generics.

**Table 1:** Characteristics of study subjects.

	Without latecomers *n* = 13	With latecomers *n* = 15
Nationality		
Domestic manufacturer (*n* = 15)	6	9
Overseas manufacturer (*n* = 13)	7	6
Route of administration		
Oral form (*n* = 18)	5	13
Other forms (*n* = 10)	8	2
Number of generics		
Five or fewer generics (*n* = 17)	13	4
Six or more generics (*n* = 11)	0	11

### Penetration curves of generics

**Figure 1:**
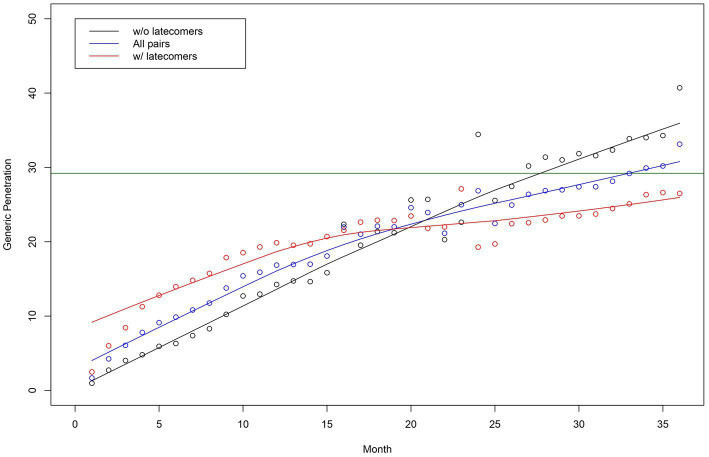
Generic penetration curves for all originator-generic pairs—pairs without and with latecomers. Note: The horizontal line in green indicates the estimated market share of generics using a logistic growth model.

Figure 1 depicts the penetration curves of the generics in all pairs, both with and without latecomers. This figure presents the market share of generics, the time to saturated market share, and the speed of generics’ penetration. The saturated market share of generics was 30%, and the time to market share saturation was approximately 33 months. The market share saturation was higher for pairs without latecomers than those with latecomers. The observed speed of those pairs with latecomers increased immediately after the market entry of the first generics and decreased continuously throughout the study period. The speed of pairs without latecomers was slower than those with latecomers immediately after the market entry of the first generics. These slower penetration speeds were sustained throughout the study period.

**Figure 2:**
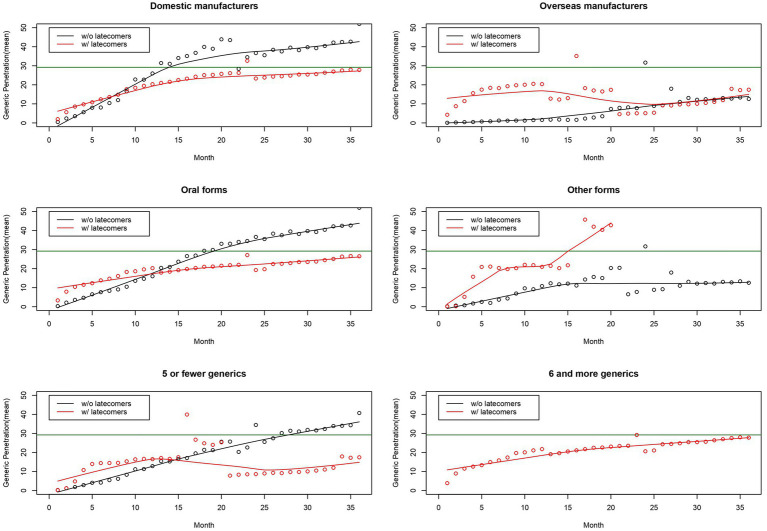
Generic penetration curves separated by nationality, a route, and number of generics. Note: The horizontal line in green indicates the estimated market share of generics using a logistic growth model.

Figure 2 describes the penetration curves of generics separated by nationality, route, and number. The penetration curves of generics varied by factors. First, some curves were stable, while other curves fluctuated. The curves of pairs originating from overseas manufacturers and pairs with five or fewer generics tended to fluctuate. The curves of pairs originating from domestic manufacturers, those administered in oral forms, and those with six or more generics were stable. Second, the shapes of the curves varied depending on the presence of latecomers. In the early stages, the observed market share of pairs with latecomers was higher than that of pairs without latecomers. The curves of pairs with latecomers and those without latecomers intersected. The observed market share of pairs without latecomers was higher than those with latecomers at later stages.

### Saturated market share of generics

Table 2 presents the saturated market share of generics, estimated by a logistic growth model using the nonlinear least squares method. The table’s first, second, and third columns display the estimated market shares of all pairs, those without latecomers, and those with latecomers, respectively. Their estimated market shares were 29.21, 35.32, and 23.45%. The estimated market shares of pairs with latecomers were divided into the market shares of first movers and latecomers. Their estimated market shares were 18.34 and 5.11%.

**Table 2:** Estimated generics market shares using the logistic growth model.

			With latecomers		
	All	Without latecomers	All generics	First generics	Latecomers
	29.21***	35.32***	23.45***	18.34***	5.11
Nationality					
Domestic	32.24***	40.08***	26.33***	19.96***	6.37
Overseas	Not significant	14.08***	16.59***	15.62***	0.97
Route of administration					
Oral	32.83***	43.35***	23.43 ***	17.74***	5.69
Other	16.65***	14.78***	Not significant	33.95***	Not available
Number of generics					
Five or fewer	31.36***	35.32***	17.65***	15.95***	1.70
Six or more	25.16***	–	25.16***	18.87***	6.29

***p < 0.001.

The estimated market shares of generics varied by factors. First, the estimated market shares of pairs in oral and other forms were 32.83 and 16.65%. Second, increased number of generic brands were not linked to increased generics’ market shares. The estimated market shares of pairs with five or fewer generics and those with six or more generics were 31.36 and 25.16%. Third, the estimated market share of pairs originating from domestic manufacturers was 32.24%. The estimated market shares of pairs originating from overseas manufacturers were non-significant. However, their market shares looked lower than those from domestic manufacturers. Finally, the estimated market share of latecomers was less than 7% in all groups. The market shares of latecomers were low for pairs originating from overseas manufacturers (0.97%) and those with five or fewer generics (1.70%). In contrast, the market share of latecomers was comparatively high for pairs originating from domestic manufacturers (6.37%), those in oral forms (5.69%), and those with six or more generics (6.29%).

## Discussion

This study provides a detailed description of generic penetration after introducing the patent linkage system. We categorized generics into first movers and latecomers, described the penetration curves of generics, and estimated the saturated market share of generics, including first movers and latecomers. Our analyses presented generic market dynamics and first mover market share advantages under the patent linkage system. Findings from this study have the potential to provide insights into the generic market and the rationale of exclusivity for the first generics as a valid component of the patent linkage system.

### Understanding the overall generic market

Promoting the use of generics is essential to managing pharmaceutical expenditure and enhancing access to medicines. Several studies have analyzed generic or biosimilar markets in many countries. However, few studies have evaluated generic uptake immediately after the first generic entrance (13).

In the United States, generics have promptly penetrated the market. Mean generic uptake was 66% in the first year and 83% in the second year of the first generic entrance (13). A more recent study reported that the saturated market share of generics was 80%, and the time to saturation was approximately 12 months (14). Various studies on biosimilar uptake and their market competition have been reported (15–18). Biosimilar uptake is slower than generic penetration, and biosimilar uptake in the United States has been delayed compared to that in other countries (19–21). However, biosimilar penetration has accelerated in the United States. Biosimilars launched in 2020 reached a 70% market penetration within less than 12 months, while those biosimilars launched in 2018 took 36 months to achieve a 70% penetration (22).

Generic penetration highly depends on context (13, 14, 20, 21, 23–25). The literature reports several factors associated with generic uptakes (26, 27). Saturated generic penetration varies among therapeutic categories (25). Originator’s market size is a crucial determinant of generic penetration (25–27). Generic penetration in high-volume markets was higher than that in low-volume markets (13, 14). In contrast, injectable drugs and markets with a limited number of generic brands tend to show lower degrees of penetration (26). Furthermore, generic penetration is influenced by government policies on the pricing and promotion of generics (28, 29). Substantial price discounts initiated by generic manufacturers would be linked to increased generic penetration. The degree of generic penetration significantly declines 12 months after the market entrance of the first generic, indicating that the penetration speed within 12 months determines the saturated penetration (21, 22, 30).

### Insights into the generic market in South Korea

This study presents interesting findings on the dynamics of generic penetration, including the saturated market share, the time to saturation, and the speed of penetration. First, the saturated market share of generics was 30%, and the time to saturation was approximately 33 months. Compared to the generic and biosimilar uptake in the United States, generic penetration in South Korea is delayed and limited. Government policies on the pricing of generics explain these interesting observations. The prices of originators and their generics were set under the “same compound is the same price” principle (31). In particular, the statutory pricing scheme determined the maximum reimbursed prices of originators and their generics. Note that manufacturers can voluntarily discount the price of their products under the maximum reimbursed price. However, the price of originators and their generics had been stuck to the maximum reimbursed price determined by the pre-determined scheme (32). Even latecomers at the market had been reluctant to initiate price competition (33).

Second, the shapes of generic penetration were context-dependent. However, the existence of latecomers was associated with the decreased penetration speed over time and decreased market share of generics. In a similar vein, markets with limited numbers of generic brands were shown to have higher levels of generic penetration. We observed decreasing slopes in the curves for pairs with latecomers after 12 months, which is consistent with other countries (21, 22, 30). However, the penetration curves of pairs without latecomers did not notably change. Instead, their market penetration continuously increased during the observation period. These findings provide interesting evidence regarding the market dynamics directly after the entrance of the first generic.

Pairs without latecomers might indicate one of two things—either that the development of generics was complicated or that the expected profits of latecomers were not sufficiently high (34, 35). Entering generics into this market might guarantee brand loyalty from physicians. Accordingly, first-generation generics would be less likely to pursue marketing activities to enhance their market share in the early stages, and their market share would continue to increase over the long term. Conversely, pairs with latecomers might indicate either that the development of generics was not complicated or that the expected profits of latecomers were sufficiently high (34, 35). Entering generics into this market might not guarantee brand loyalty. The first generics would be more likely candidates for marketing activities to enhance their market share in the early stages. Their initial market share would increase rapidly. In contrast, latecomer market shares were shown to be marginal in later stages, which might be associated with further latecomer disadvantages (36–38). These findings are consistent with the previous study, indicating that more generic brands were not associated with increased generic penetration (27).

Third, we consistently observed the first mover market share advantages or latecomers’ disadvantages under the patent linkage system. The terms “first movers” and “latecomers” can be interpreted in two ways. First, looking at the overall market, originator drugs could be defined as first movers, leaving generics to be defined as latecomers. Second, generics that enter the market first can be defined as first movers, leaving the remaining generics to be defined as latecomers. This study adopts the second definition in measuring the advantages of first movers or disadvantages of latecomers. Pairs originating from overseas manufacturers had marginal market shares for latecomers, while pairs originating from domestic manufacturers had increased market shares for latecomers. We also found that pairs originating from overseas manufacturers presented marginal generic penetration, while pairs originating from domestic manufacturers showed increased generic penetration. These observations indicate that the advantages of first movers and the disadvantages of latecomers apply to both definitions of the terms. Marginal first generic penetration is linked to a marginal latecomer market share, while substantial first generic penetration is associated with an increased latecomer market share. The market share of first generics derives from the dynamics of the specific drug market, and they could be used to predict the market share of latecomers.

### The rationale of exclusivity for the first generics as components of the patent linkage system

South Korea introduced the linkage system in 2012 after signing the KORUS (2, 3). Delayed generic entry and the high prices of originators are pivotal issues in introducing the patent linkage system (4–7). To counter a stay of generics, the government established nine-month exclusivity to incentivize generic manufacturers to challenge the validity of patents and to enter the market expediently (39).

Similar to the case in South Korea, many developing countries have experienced difficulties in introducing the patent linkage system (40). Many researchers have been concerned that patent rights are private, and private rights need to be protected privately (2, 5). Some researchers have argued that the patent linkage system, which protects private rights through a national regulatory process, should not be implemented in developing countries. We agree with these concerns and arguments. However, introducing the patent linkage system can only be avoided in some cases. China (41) and Taiwan (42) have recently adopted the system. Member countries in the Comprehensive and Progressive Agreement for Trans-Pacific Partnership (CPTPP), such as Brunei, Singapore, New Zealand, Chile, and Vietnam, might introduce the patent linkage system relatively soon, as the agreement requires the linkage system (43). The lessons learned from South Korea demonstrate a rationale for the first generic exclusivity as a valid component of the patent linkage system.

### Study strengths and limitations

This study has several strengths. First, we analyzed the dynamics of generic penetration in South Korea immediately after the first generic entrance. Originators with corresponding generics granted the first generic exclusivity from 2015 to 2020 were identified, and their monthly sales data until December 2020 were obtained. Second, we leveraged the first generic exclusivity system to distinguish generics into first movers and latecomers. The first generic exclusivity system, which guarantees 9 months of exclusivity for first generics, offered a quasi-experimental study design to analyze the first-mover advantages in the pharmaceutical market. Third, we captured the penetration curves of generics beyond their saturated market shares. We analyzed the time to market saturation and the speed of penetration to understand market dynamics following the entrance of generics.

This study has several limitations. First, the total number of investigated pairs was 28, which needs to be increased for generalizing study findings. Similarly, some of our logistic growth models, as distinguished by various variables, were unstable in estimating the saturated penetration of generics. Second, this study analyzed generic penetration in the South Korean market, in which the penetration of generics has been reported as marginal. Thus, generalizing our study findings to other markets where generic penetration is quite active should be cautiously pursued. Third, this study found an association between the existence of latecomers and generics market penetration. However, any potential causal relationship between the two variables should be studied further. For instance, the nonexistence of latecomers might be influenced by the anticipation of low generic penetration.

## Conclusion

This study provides evidence of the dynamics of generic markets and the rationale of exclusivity for the first generics. The generic uptake in South Korea is delayed, limited, and context-dependent. However, the first generic market share advantages were consistently observed even after introducing the patent linkage system. The lessons learned from South Korea demonstrated that a period of exclusivity could be used to provide an economic incentive for early generic entrance, even under the patent linkage system.

## Data availability statement

The data that supports the findings of this study are available from the corresponding author, upon reasonable request.

## Author contributions

K-BS developed the concept of the manuscript, undertook the analysis, and wrote the manuscript.

## Funding

This work was supported by the research fund of National Research Foundation (NRF-2022R1F1A1071338).

## Acknowledgments

We acknowledge research support from the Ministry of Food and Drug Safety.

## Conflict of interest

The author declares that the research was conducted in the absence of any commercial or financial relationships that could be construed as a potential conflict of interest.

## Publisher’s note

All claims expressed in this article are solely those of the authors and do not necessarily represent those of their affiliated organizations, or those of the publisher, the editors and the reviewers. Any product that may be evaluated in this article, or claim that may be made by its manufacturer, is not guaranteed or endorsed by the publisher.

## Abbreviations

CPTPP: Comprehensive and Progressive Agreement for Trans-Pacific Partnership
KORUS: Korea United States Free Trade Agreement
