# Supplementary figures and images for: Does the first generic exclusivity system provide an economic incentive for early generic entrance under the patent linkage system?
Source: Front Public Health. 2023 Aug 3;11:1120729. doi: 10.3389/fpubh.2023.1120729 (PMC10435867; doi:10.3389/fpubh.2023.1120729)

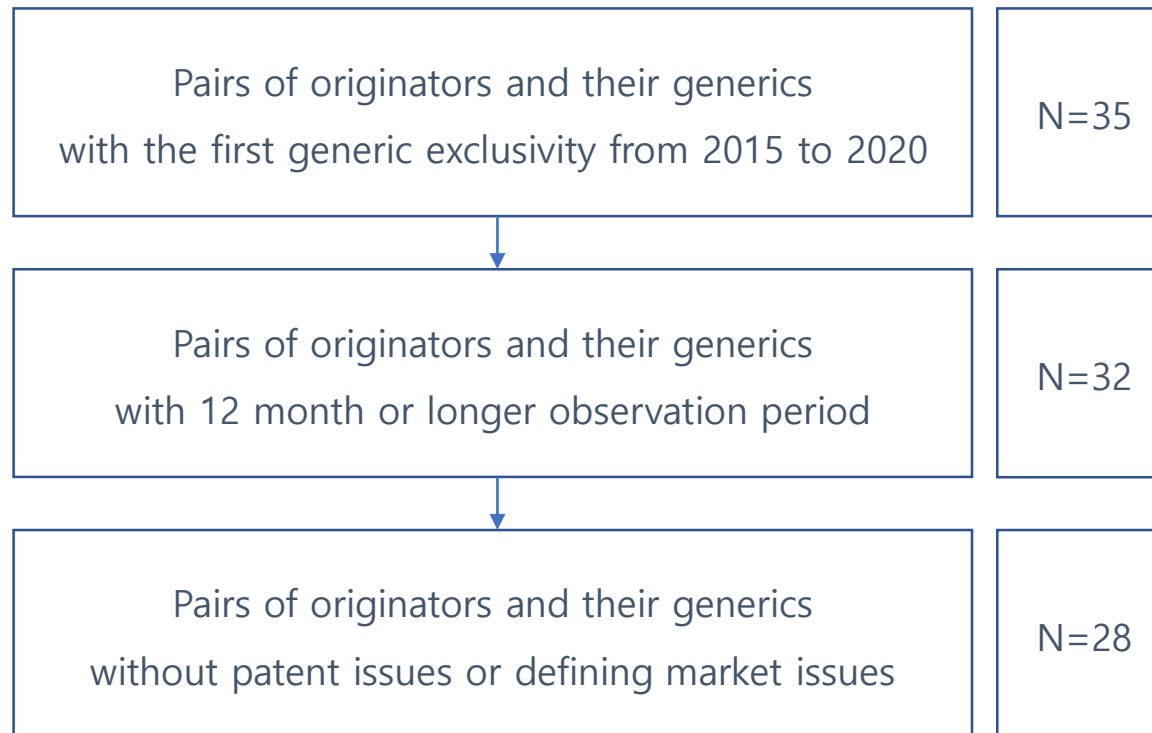

Supplement: Supplementary file 1 [file Image_1.pdf]
