# Supplementary material for: Does the first generic exclusivity system provide an economic incentive for early generic entrance under the patent linkage system?
Source: Front Public Health. 2023 Aug 3;11:1120729. doi: 10.3389/fpubh.2023.1120729 (PMC10435867; doi:10.3389/fpubh.2023.1120729)
Supplement: Supplementary file 2 [file Presentation_1.pptx]

## Slide 1
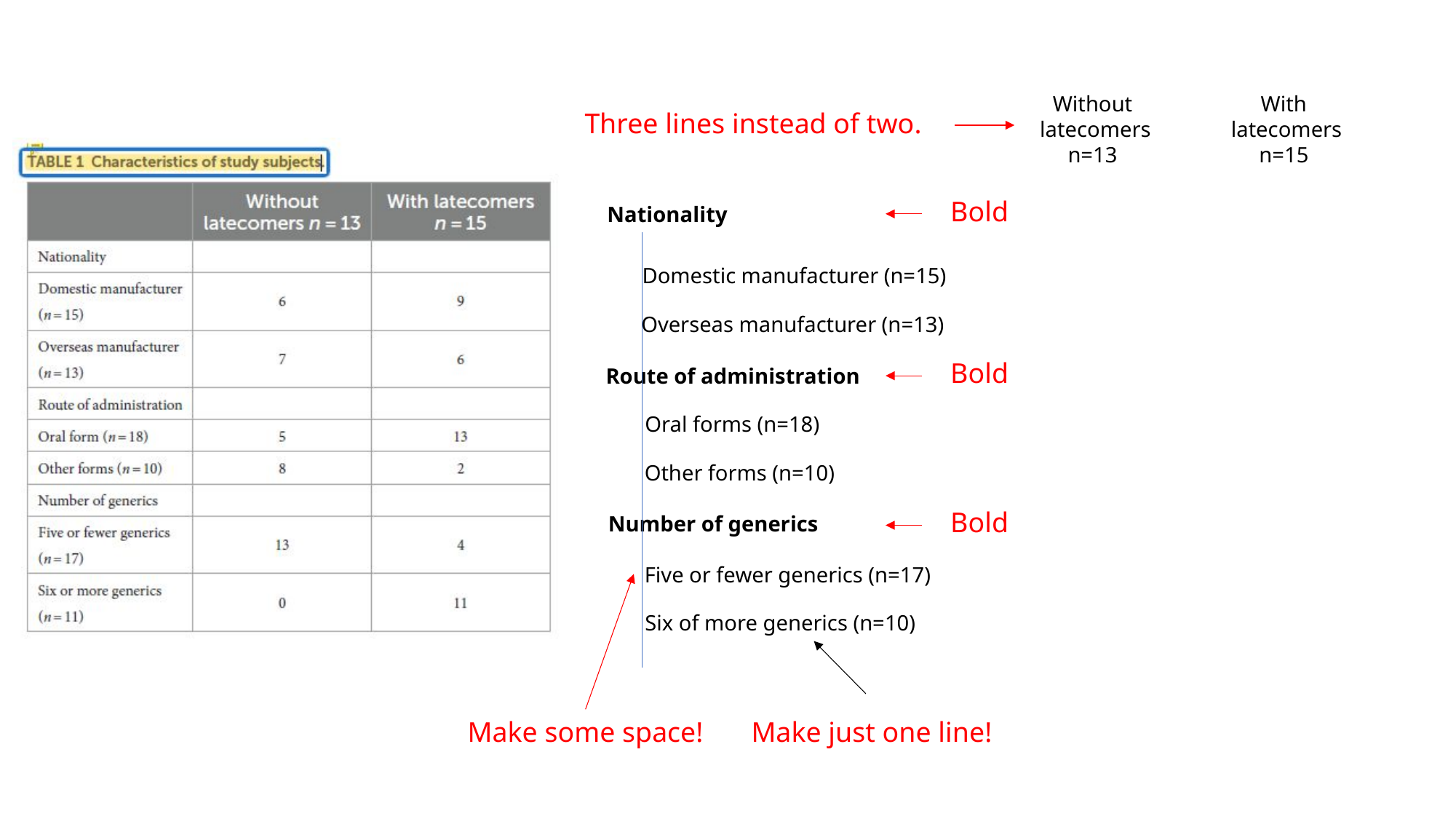

Without
latecomers
n=13
With
latecomers
n=15
Three lines instead of two.
Bold
Nationality
Domestic manufacturer (n=15)
Overseas manufacturer (n=13)
Bold
Route of administration
Oral forms (n=18)
Other forms (n=10)
Bold
Number of generics
Five or fewer generics (n=17)
Six of more generics (n=10)
Make some space!
Make just one line!

## Slide 2
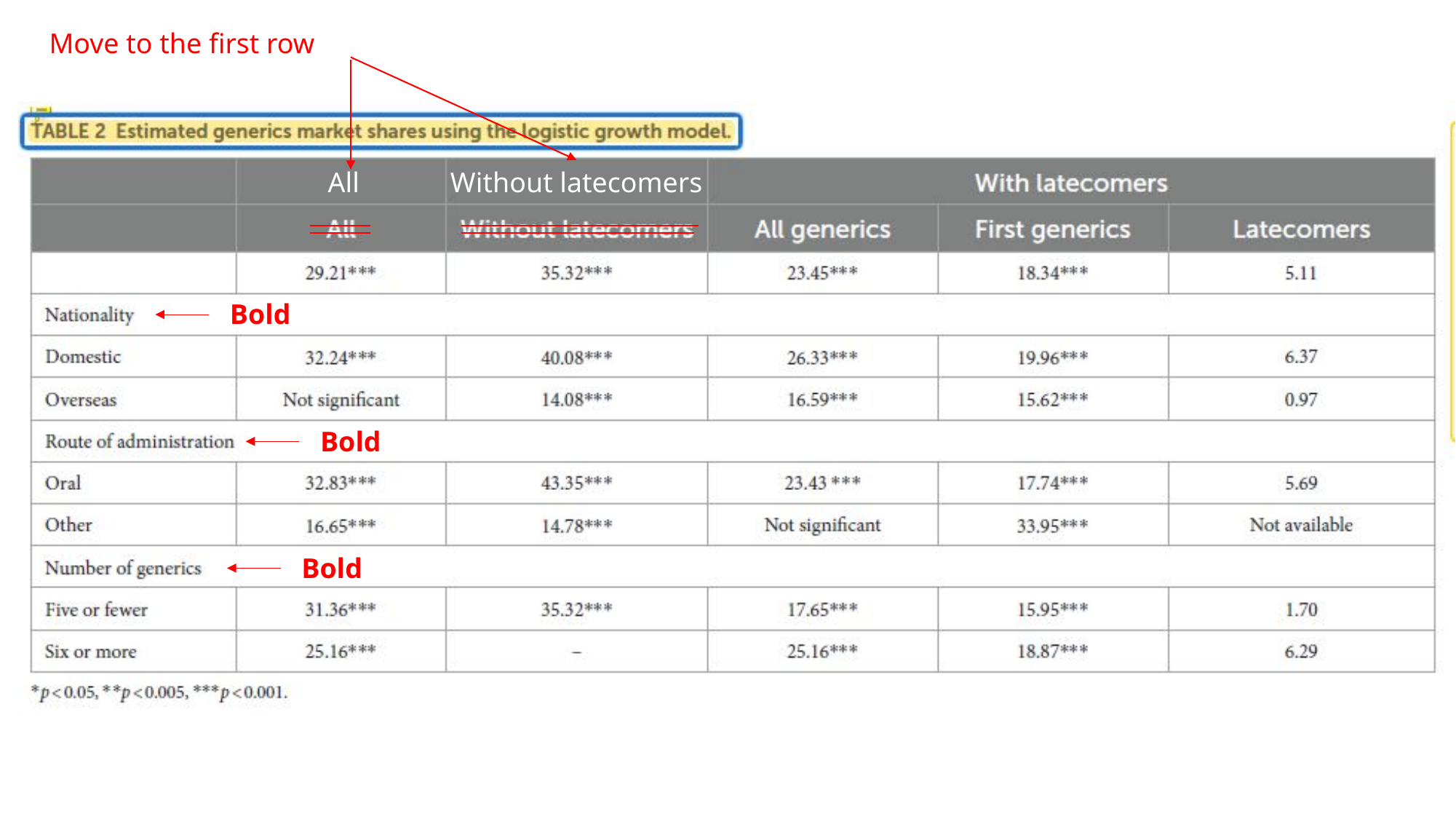

Move to the first row
All
Without latecomers
Bold
Bold
Bold
